# Partial directed coherence analysis of resting-state EEG signals for alcohol use disorder detection using machine learning

**DOI:** 10.3389/fnins.2024.1524513

**Published:** 2025-01-10

**Authors:** Ainul Khairiyah Mohd Nazri, Norashikin Yahya, Danish M. Khan, Noor'Izni Zafirah Mohd Radzi, Nasreen Badruddin, Abdul Halim Abdul Latiff, Mohammed J. Abdulaal

**Affiliations:** ^1^Maintenance Department, PETRONAS Gas Berhad, Kerteh, Terengganu, Malaysia; ^2^Centre for Intelligent Signal and Imaging Research (CISIR), Electrical and Electronic Engineering Department, Universiti Teknologi PETRONAS, Bandar Seri Iskandar, Perak, Malaysia; ^3^Department of Data Science and Artificial Intelligence, School of Engineering and Technology, Sunway University, Petaling Jaya, Selangor, Malaysia; ^4^Centre for Subsurface Imaging, Department of Geosciences, Universiti Teknologi PETRONAS, Bandar Seri Iskandar, Perak, Malaysia; ^5^Center of Excellence in Intelligent Engineering Systems (CEIES), Department of Electrical and Computer Engineering, Faculty of Engineering, King Abdulaziz University, Jeddah, Saudi Arabia

**Keywords:** alcohol use disorder (AUD), effective connectivity, electroencephalography (EEG), partial directed coherence (PDC), support vector machine (SVM)

## Abstract

**Introduction:**

Excessive alcohol consumption negatively impacts physical and psychiatric health, lifestyle, and societal interactions. Chronic alcohol abuse alters brain structure, leading to alcohol use disorder (AUD), a condition requiring early diagnosis for effective management. Current diagnostic methods, primarily reliant on subjective questionnaires, could benefit from objective measures.

**Method:**

The study proposes a novel EEG-based classification approach, focusing on effective connectivity (EC) derived from resting-state EEG signals in combination with support vector machine (SVM) algorithms. EC estimation is performed using the partial directed coherence (PDC) technique. The analysis is conducted on an EEG dataset comprising 35 individuals with AUD and 35 healthy controls (HCs). The methodology evaluates the efficacy of connectivity features in distinguishing between AUD and HC and subsequently develops and assesses an EEG classification technique using EC matrices and SVM.

**Result:**

The proposed methodology demonstrated promising performance, achieving a peak accuracy of 94.5% and an area under the curve (AUC) of 0.988, specifically using frequency bands 29, 36, 45, 46, and 52. Additionally, feature reduction techniques applied to the PDC adjacency matrices in the gamma band further improved classification outcomes. The SVM-based classification achieved an accuracy of 96.37 ± 0.45%, showcasing enhanced performance through the utilization of reduced PDC adjacency matrices.

**Discussion:**

These results highlight the potential of the developed algorithm as a robust diagnostic tool for AUD detection, enhancing precision beyond subjective methods. Incorporating EC features derived from EEG signals can inform tailored treatment strategies, contributing to improved management of AUD.

## 1 Introduction

The economic and social consequences of alcohol use disorder (AUD) is one of the significant issues in today's society. It has a significant impact on the brain functionality, especially with heavy and chronic use. It can impair cognitive skills and alter brain structure by shrinking the white and gray matter of the brain, which leads to balance, speech, and coordination problems (Nunes et al., 2019). The severity of the effects varies depending on the amount and frequency of the consumption and the duration of alcohol use. Furthermore, alterations in particular regions of the brain can impact the likelihood of developing AUD (Lim and et al., 2021). AUD is a neurological condition that hinders the capacity to cease or regulate alcohol intake in a moderate manner (Jarcuskova et al., 2024). Hence, early diagnosis of AUD is one of the critical factors in successful treatment and therapy management of AUD subjects before it prolongs to a critical stage. A healthcare provider or addiction specialist will evaluate the symptoms and provide a proper diagnosis. The diagnosis identifies the signs and symptoms of AUD before it progresses to more severe stages. Early detection and treatment can increase the likelihood of recovery by preventing further the development of AUD (Masroor et al., 2024).

Mental health professionals use the Diagnostic and Statistical Manual of Mental Disorders, Fifth Edition (DSM-5) to diagnose and categorize psychiatric disorders, including AUD (Källmén et al., 2019). It is divided into sections that list each disorder's diagnostic standards and codes. In order to aid in the diagnostic process, it also contains information on differential diagnosis, cultural considerations, and assessment tools. However, it has also received criticism for its reliance on categorical diagnoses and disregard for contextual and personal factors that could have an impact on mental health. Thus, professionals often use DSM-5 with other assessment tools to provide a comprehensive evaluation of a person's mental health.

In this work, effective connectivity (EC) will be used to classify AUD subjects from healthy controls (HCs). The EC of a brain node, which is derived from a model of neuronal integration that specifies the mechanisms of neuronal coupling, is the influence that a node has over another under a network model of causal dynamics. Numerous research investigations have explored the effects of AUD on neural connectivity, employing neuroimaging methods like electroencephalography (EEG) and functional magnetic resonance imaging (fMRI) (van Oort et al., 2023). The precuneus and lateral parietal regions of the brain, which are involved in the integration of information relating to the perception of the environment, were found to have reduced EC in individuals with AUD, according to a study (Khan et al., 2021a). Another study demonstrated that individuals with AUD exhibit less intense EC in the Default Mode Network (DMN) regions compared to the healthy individuals (Khan et al., 2021c). Low performance on the task and the severity of alcohol consumption were both strongly associated with the decreased connectivity. Hence, analyzing EEG-recorded brain activity patterns can reveal the relationships between different brain regions and their causal effects on other regions. Overall, these studies collectively suggest that EC can be significantly impacted by AUD, which contributes to the behavioral and cognitive deficits associated with the disorder.

Finally, recent advancements in neuroscience research have placed an increasing emphasis on network patterns of brain dynamics (Hou and et al., 2022; Khan et al., 2023; Shen et al., 2024). Hence, EC matrices are used for training and testing convolutional neural network (CNN) for the best classification result. The estimation of EC as a connectivity structure for classification can be done using mathematical techniques such as partial directed coherence (PDC), or dynamic causal modeling (DCM). Then, the 3D EC connectivity structure is transformed into 2D images, which serve as input to the CNN (Khan et al., 2021c).

The role of EC as an input to CNN offers a more precise analysis of the EEG data by distinguishing between direct and indirect connections and identifying the precise direction of information flow. Additionally, research has demonstrated that EC of EEG signals in the frequency bands could serve as a promising biomarker for the diagnosis of AUD (Khan et al., 2021c). Thus, the capabilities of EC in carrying the features of AUD subject and its performance for diagnosis will be investigated. The objectives of this study is to develop a robust and accurate machine learning algorithm for AUD detection using EC matrices derived from resting-state EEG signals.

This paper employs PDC adjacency matrices derived from EEG recordings to classify individuals with AUD and HC. These matrices serve as input for a machine learning model, SVM. The adjacency matrices are created through PDC calculations across 64 frequency bands, evenly divided within the range of 0.1 Hz to 40 Hz.

The paper is structured in the following manner: Section 2 covers related studies on brain connectivity and its application in classifying various neurological conditions. Section 3 focuses on the methods utilized to create and assess an EEG classification algorithm for AUD detection, involving PDC adjacency matrices and SVM. The result and discussion of the research are explained in Section 4. Lastly, Section 5 will conclude this paper.

## 2 EEG brain connectivity for detection of neurological conditions—its related studies

The activity of brain cells communicating via electrical impulse can be measured and recorded by EEG and is shown on recording in the forms of wavy lines (Khan et al., 2021b; Al-Hiyali et al., 2019). The most common application of an EEG is to identify and evaluate epilepsy (Wang et al., 2020; Wen et al., 2022), a brain disorder that frequently results in recurrent seizures. A lot of past research utilizes EC from EEG recordings in analyzing neurological disorders such as depression, autism, and epilepsy (Khan et al., 2022; Parker and et al., 2018; Rolls and et al., 2018). It shows that EC has a good potential in determining AUD and the methods could be extended by using CNN. EEG can aid in the diagnosis by supplying more details about the many types of epilepsy, its various therapies, and the underlying triggers. Similarly, the recorded EEG data of human brain can also be used to identify other disorders such as the AUD (Anuragi and Singh Sisodia, 2019; Khosla et al., 2020). In order to extract and analyze the desired information from the EEG signal, the analysis of EEG will make use of mathematical signal analysis techniques and computing technologies.

Deep learning techniques have proven beneficial in classifying neurophysiological signals, including EEG, for the diagnosis of AUD. A study applied a deep CNN to EEG signals and calculated the EC within the DMN brain regions, achieving an impressive overall accuracy of 92.7% for AUD classification (Khan et al., 2021c).

Furthermore, the ability of wavelet scattering techniques (WST) based features extracted from multichannel EEG signals were explored for AUD classification using SVM classifier (Buriro and et al., 2021). According to articles, WST-based shows a convincing alternative to CNN. This reflects WST's characteristics, which include high-frequency information durability, susceptibility to signal deformation, and connectivity to the occipital and parietal regions.

Then, in another study for automated AUD identification, SVM with rabial basis function (SVM-RBF) kernel was developed (Anuragi et al., 2020). The FBSE-EWT (Fourier-Bessel series expansion with based empirical wavelet transform) was used to derive entropy values from 112 EEG recordings of healthy and AUD subjects. FBSE-EWT decomposes EEG signals into sub-band signals with 20 significant extracted features. Using “Leave-one-sample-out” cross validation for performance evaluation, it achieves an accuracy of 98.8%.

On the other hand, two deep learning techniques were compared for the classification of AUD EEG signals (Farsi et al., 2021). The experiment consists of 77 subjects diagnosed with AUD and 45 healthy participants. Firstly, the extracted features of EEG using Principal Component Analysis (PCA) based feature extraction are used as input to “long term short memory” (LTSM) and is compared to second methods which the raw EEG data is directly fed to Artificial Neural Network (ANN) as an input for the classification of AUD. The experimental findings show that the second technique produces an average classification accuracy of 93% using a 10-cross validation strategy, whereas the first method achieves an accuracy of 86%. Therefore, deep learning and machine learning approaches have demonstrated potential in effectively categorizing EEG signals for diagnosing AUD. Its robust and data-driven approach enables it to identify subtle differences between EEG recordings of individuals with AUD and HCs, by automatically extracting relevant features and learning from large datasets.

In Dafters et al. (1999) investigate whether there is a correlation between quantitative EEG variables (spectral power and coherence) and cognitive/mood variables, and level of prior use of methylenedioxymethamphetamine (MDMA or Ecstasy). Pearson correlation analyses were employed to investigate the association between various measures and the subjects' consumption of MDMA over the past 12 months. The use of MDMA showed a positive correlation with absolute power in the alpha frequency, where alpha power is known to have an inverse relationship with mental function. Additionally, decreased coherence levels, linked to dysfunctional connectivity, have been previously associated with brain disorders such as dementia.

To investigate whether the neuronal network undergoes dynamic changes before and during the transition to an EEG epileptic discharge, Varotto et al. (2012) estimate EEG connectivity patterns in photosensitive (PS) patients with idiopathic generalized epilepsy. The results revealed that individuals with PS exhibit abnormal EEG hyperconnectivity, particularly affecting the anterior cortical regions. This hyperconnectivity pattern is observed both during resting conditions and intermittent photic stimulation (IPS).

The PDC technique of Baccalá and Sameshima (2001) is a widely used method for the problem. The three subjects of Pascual-Marqui et al. (2014) are: (1) To show that the PDC can misrepresent the frequency response under plausible realistic conditions, thus defeating the main purpose for which the measure was developed; (2) To provide a solution to this problem, namely the isolated effective coherence (iCoh), which consists of estimating the partial coherence under a multivariate autoregressive model, followed by setting all irrelevant associations to zero, other than the particular directional association of interest; and (3) To show that adequate iCoh estimators can be obtained from non-invasively computed cortical signals based on exact low resolution electromagnetic tomography (eLORETA) applied to scalp EEG recordings.

Networks of functional and effective connectivity were described using a spatial filter approach called the dynamic imaging of coherent sources (DICS) followed by the renormalized partial directed coherence (RPDC) (Muthuraman et al., 2015). The suggested approach demonstrates efficacy in generating efficient features for the detection of Motor Imagery (MI) tasks. It holds significant promise for application in Brain-Computer Interface (BCI) systems (Liang et al., 2016).

The aim of Japaridze et al. (2016) was to investigate the neuronal networks underlying background oscillations of epileptic encephalopathy with continuous spikes and waves during slow sleep (CSWS). The examination of information flow within this network indicates that the medial parietal cortex, the precuneus, and the thalamus function as central hubs, directing information flow to other areas, notably the temporal and frontal cortex. The pivotal roles of the precuneus and thalamus in the hierarchical organization of the network associated with the background EEG suggest the importance of vigilance fluctuations in the generation of CSWS. The high temporal resolution of the EEG is utilized to estimate the EC within the DMN (Khan et al., 2021a). The differences in causal effects within the DMN between control subjects and individuals with AUD exhibit a noticeable correlation with symptoms commonly linked to AUD. These symptoms include cognitive and memory impairments, executive control issues, and attention deficiencies (Balconi et al., 2024). In Saeedi et al. (2021) propose an EEG-based deep learning framework that automatically discriminates MDD patients from HCs. The fusion of CNN-LSTM architecture effectively captures both spatial and temporal relations within brain connectivity.

Phang et al. (2019) propose a deep CNN framework for classification of EEG-derived brain connectome in schizophrenia (SZ). The framework combines various connectivity features that improves performances over single-domain CNN. Event-related potentials were collected from 45 SZ patients and 30 HCs during a learning task, and then a combination of PDC effective and phase lag index (PLI) functional connectivity were used as features to train a SVM classifier with leave-one-out cross-validation for classification of SZ from HCs (Zhao et al., 2021). The results suggest that the majority of the chosen EC features are significantly increased in patients with SZ compared to HCs, whereas all selected functional connectivity features demonstrate a decrease in SZ patients.

## 3 Methodology

This section provides an overview of the methodology used in this work, covering the overall approach to develop the AUD classification using EC of EEG, description of the EEG dataset, the method to determine the EC matrices from 19-channel EEG, performance measures, and lastly the approach of classification using EC and SVM.

### 3.1 Overall approach

**Figure 1:**
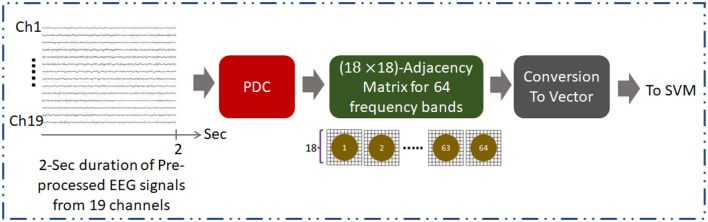
Overall methodology for development of AUD classification technique using PDC adjacency matrix (PDCaM). The PDCaM is estimated using electrode Cz as the reference, hence giving 18 × 18 PDCaM.

The general approach for development of AUD classification using EC obtained using the PDC is illustrated in Figure 1. Method based on SVM is employed for assessing the AUD classification of EC calculated at 64 bands of frequency. The performance of the classification method is evaluated based on accuracy, sensitivity, specificity, and precision metrics.

### 3.2 EEG signals dataset and preprocessing

The EEG dataset was taken from Universiti Malaya Medical Center (UMMC), Universiti Teknologi PETRONAS (UTP), and Bingkor Clinic in Sabah which was also used in Khan et al. (2021c).

EEG recordings of 35 individuals with AUD and 35 HCs are used in this study. All AUD subjects have met the criteria according to DSM-IV for alcohol addiction. UMMC and Bingkor Clinic, Kota Kinabalu, Sabah, provided the dataset of 15 HC and 35 AUD subjects. The remaining 20 HC data was obtained at UTP.

The EEG data was recorded in the morning under eye-close resting-state conditions for 5 min. Following the placement of 10–20, 19 electrodes were placed on the scalp utilizing two devices, the BrainMaster Discovery 24 EEG with 256 Hz sampling rate and amplitude in microvolts, and the Enobio system with 500 Hz sampling rate and amplitude in nanovolts.

**Figure 2:**
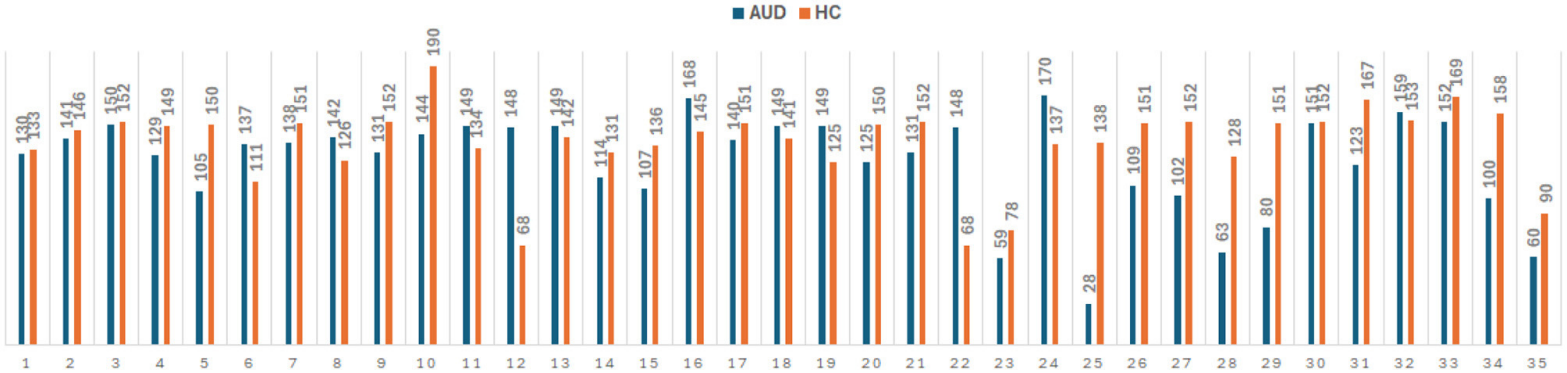
Number of PDC adjacency matrix for 35 AUD and 35 HC subjects, giving a total of 4,380 × 324 and 4,827 × 324 feature vectors for AUD and HC, respectively.

Furthermore, pre-processing steps are crucial for the analysis of EEG data to obtain accurate and reliable results. Initially, to normalize the data, EEG recordings from the Enobio system were down sampled to 256 Hz and the amplitude was converted to microvolts. Then, the normalized signals were filtered using bandpass filter (0.1–70 Hz) and notch filter (50 Hz). Artifacts removals caused by sensory motions are then applied using EEGLAB software to reduce noise and enhance the underlying brain activity. The extraction of the PDC adjacency matrices is performed on every 2-sec duration of the pre-processed EEG as illustrated in Figure 1. Figure 2 provides information on the number of PDC adjacency matrices for each AUD/HC subject.

### 3.3 Partial directed coherence adjacency matrix

PDC is a frequency domain EC technique which is based on the multivariate autoregressive model (MVAR) modeling and partial coherence (Astolfi et al., 2006). For *S* simultaneous recording of 19-channel EEG signals, the time-series representation of the signals is


(1) $$\begin{array}{l} Y\left(t\right)={\left[y_{1}\left(t\right),y_{2}\left(t\right),...,y_{S}\left(t\right)\right]}^{T}. \end{array}$$

The corresponding autoregressive model of order ρ is


(2) $$\begin{array}{l} y\left(t\right)=\overset{\rho }{\underset{\zeta =1}{\sum }}{\text{C}}_{\zeta }\text{y}\left(t-\zeta \right)+\epsilon \left(t\right). \end{array}$$

Here, $\epsilon \left(t\right)={\left[\epsilon_{1}\left(t\right),\epsilon_{2}\left(t\right),...,\epsilon_{S}\left(t\right)\right]}^{T}$ is zero-mean multivariate Gaussian white process and **C**_ζ_ is a *S*×*S* coefficient matrix, evaluate at every lag, ζ, given by


(3) $$\begin{array}{l} {\text{C}}_{\zeta }=\left[\begin{array}{ccc} c_{11}\left(\zeta \right) & \cdots & c_{1S}\left(\zeta \right)\\ \vdots & \ddots & \vdots \\ c_{S1}\left(\zeta \right) & \cdots & c_{SS}\left(\zeta \right) \end{array}\right]. \end{array}$$

If **C**(ω) is the frequency domain equivalent of coefficient matrix *C*_ζ_, then the Fourier transform of *c*_*vw*_ between 2 electrodes, *v* and *w* can be obtained as,


(4) $$\begin{array}{l} c_{vw}\left(\omega \right)=\overset{\rho }{\underset{\zeta =1}{\sum }}c_{vw}\left(\zeta \right)exp\left(-j\omega \zeta \right) \end{array}$$

The 19 × 19 PDCaM, from electrode *w* to electrode *v*, is


(5) $$\begin{array}{l} {\text{Ψ}}_{vw}\left(\omega \right)=\frac{{\bar{c}}_{vw}\left(\omega \right)}{\sqrt{{\bar{c}}_{v}^{H}\left(\omega \right){\bar{c}}_{w},\left(\omega \right)}}, \end{array}$$

where ${\bar{c}}_{vw}^{H}\left(\omega \right)$ denotes the *vw*-th elements of the matrix, ${\bar{\text{C}}}_{\zeta }$, and (.)^*H*^ represents the conjugate transpose. The MATLAB coding for the calculation of PDC adjacency matrix is available at Omidvarnia (2020).

The adjacency matrix is obtained from EC of 19-channel EEG where it encodes the connectivity of nodes. EC plays an important role as it represents the causal influence of the brain regions. This is because EC is utilized to determine the directional interdependencies of EEG data and create an asymmetric adjacency matrix, which provides a more accurate representation of the interconnections between EEG channels. Using Cz as the reference node, PDC was used to compute the PDCaM at all 64 frequency bands producing 18 × 18 dimensions of adjacency matrix from the continuous 2-sec segments of the pre-processed EEG data. Each of the 64-band frequencies has a bandwidth equal to (70–0.1) Hz/64 = 1.092 Hz. Thus, 18 × 18 dimensions represent the pairwise connectivity between 18 channels, excluding one reference channel.

**Figure 3:**
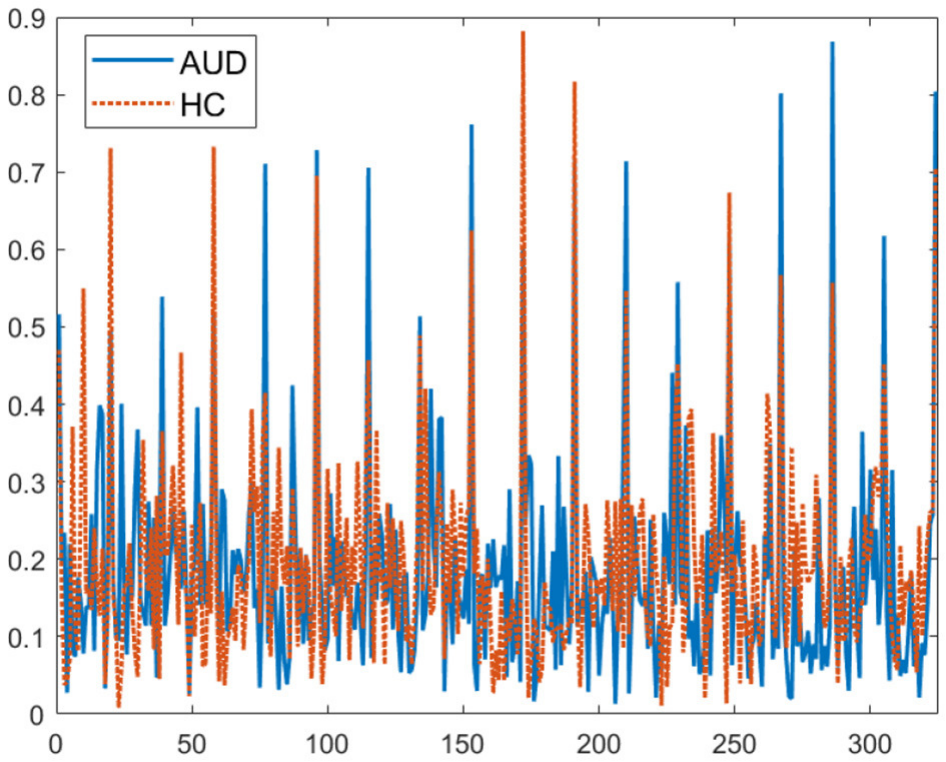
Plot of 324 PDCaM values for 2-second EEG signals from one AUD subject and one HC subject.

**Figure 4:**
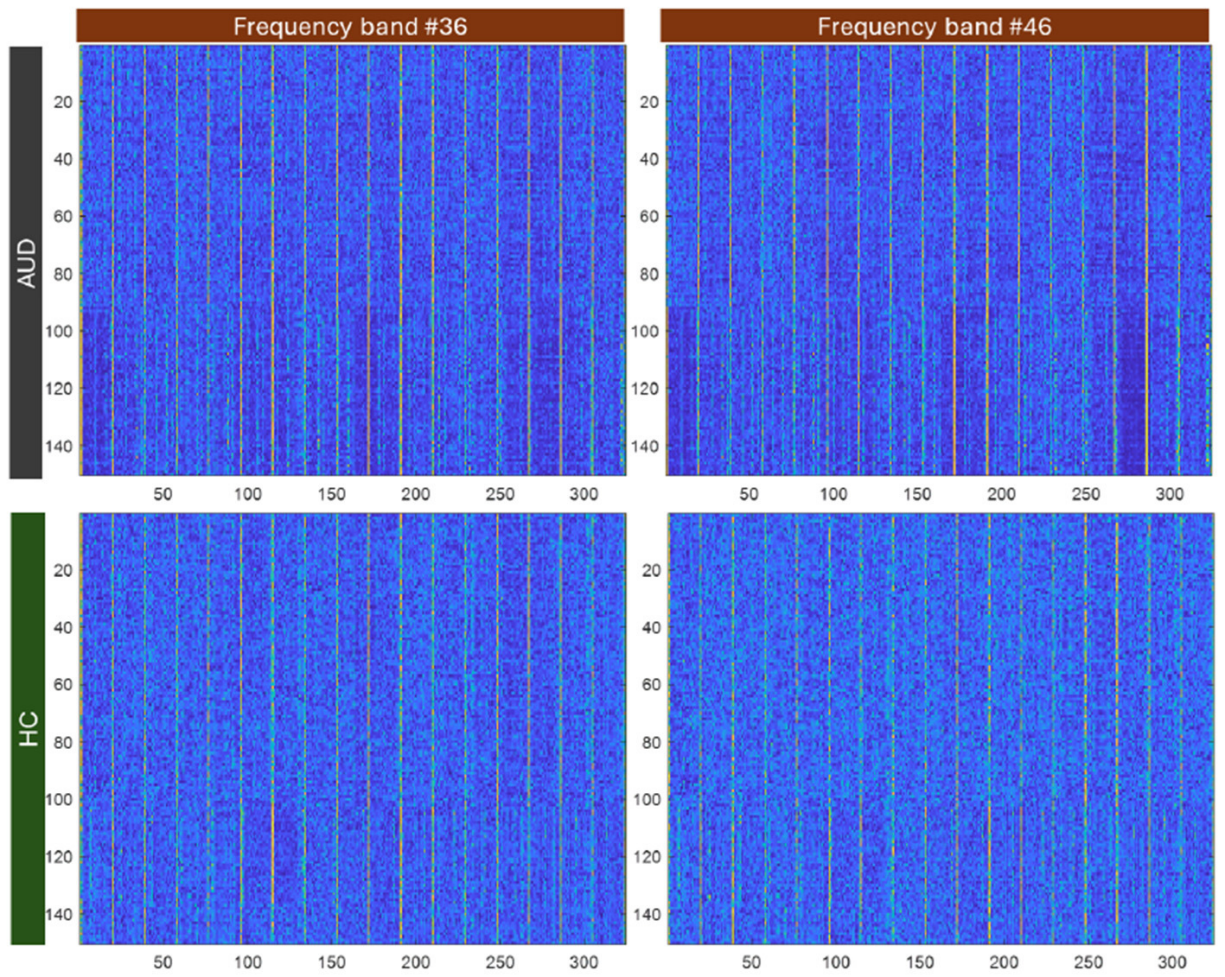
Image colorplot of 150-sample × 324-length feature vector of PDCaM for one AUD and one HC subject at 2 freqeuncy bands, 36 and 46.

For visualization, the plot of the 324-connectivity values are provided in Figure 3. These values obtained using Equation 5, are normalized to be between 0 and 1. For more meaningful visualization, 2D plots of the PDCaM for AUD and HC are prepared for two selected frequency bands, as shown in Figure 4.

In Figure 4, the PDCaM of 150 samples × 324-length feature vectors is plot for one AUD subject and HC subject and plot using a “colormap” to gain a better visualization of the data. The image are generated for 2 frequency bands, 36 and 46. As observed, the series of PDCaM values of the AUD subject has deeper blue and yellow colors than the more uniform pattern for HC subject, indicating potential differences in connectivity patterns. While the “colormap” visualization of the PDCaM may provide some initial insights, a comprehensive analysis of the EEG signals is necessary to accurately classify the subjects and draw meaningful conclusions.

### 3.4 Performance evaluation of the classification model

**Figure 5:**
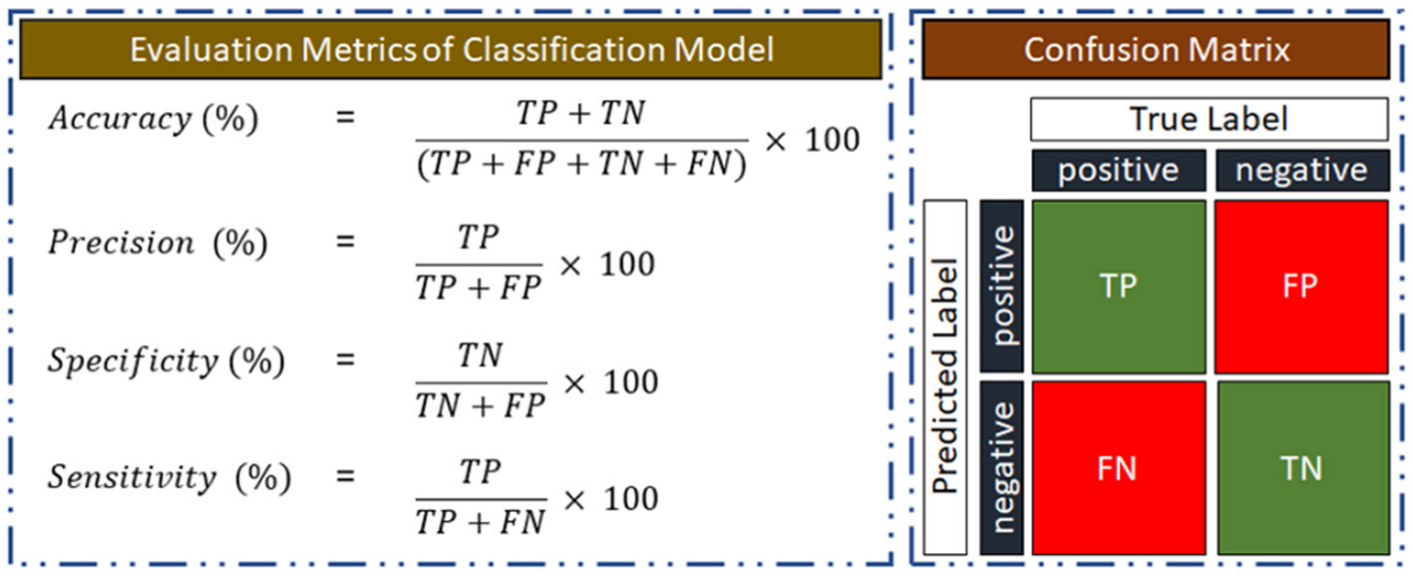
Evaluation metrics for training and testing the classification model.

Using a confusion matrix, the performance quality will be assessed depending on how well the classification model can distinguish between subjects with AUD and HC. Illustration of the confusion matrix, where AUD is the positive class is given in Figure 5. As a measure of its classification performance, the model's output will provide the number of true positives (TP), true negatives (TN), false positives (FP), and false negatives (FN). Accuracy, precision, sensitivity, and specificity performance matrices for the classification model will also be computed using the confusion matrix.

### 3.5 AUD vs. HC classification of 64-band PDCaM using SVM

**Figure 6:**
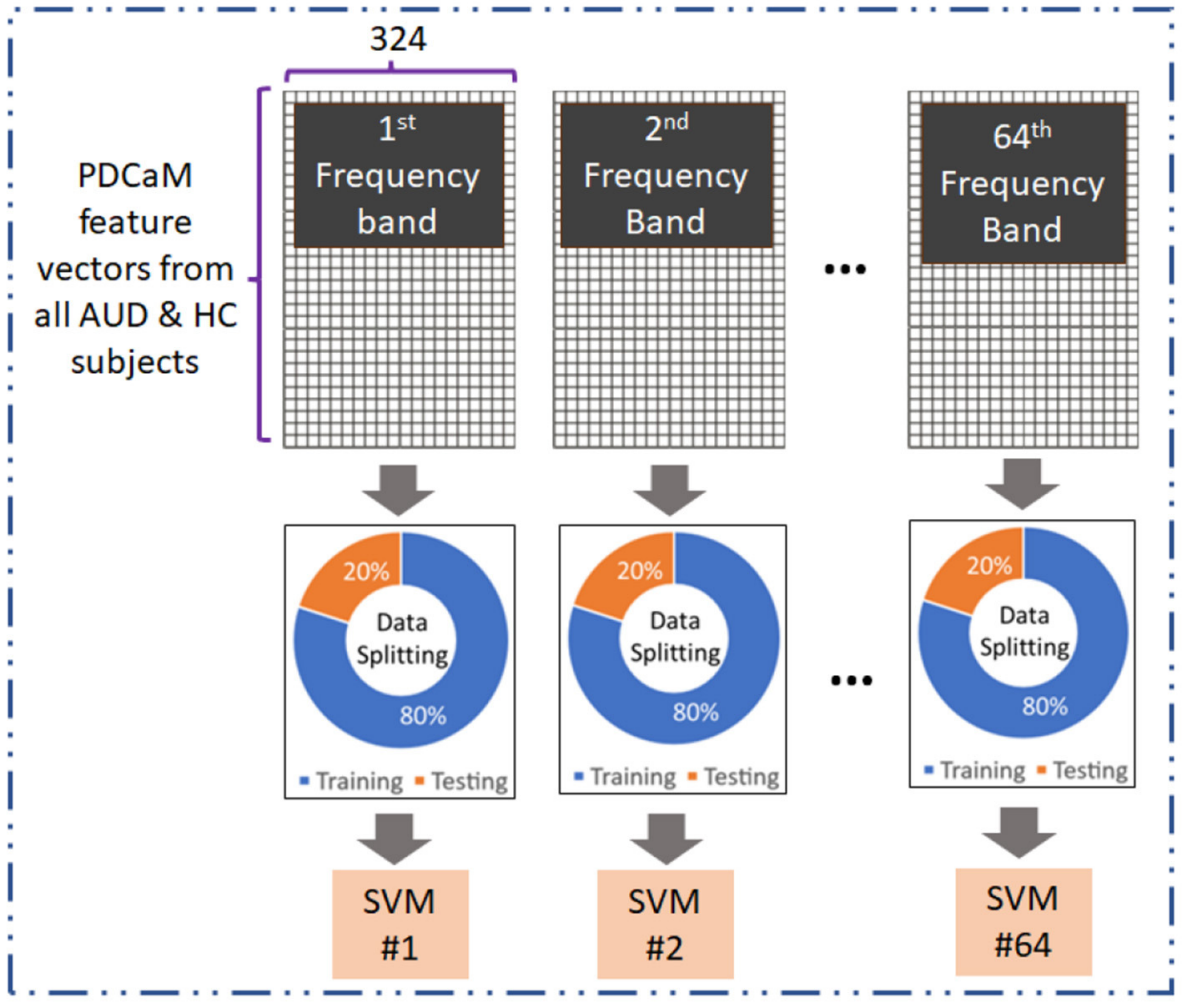
Evaluation of 64-frequency bands PDCaM in classification of AUD vs. HC using SVM.

The evaluation of the PDCaM in discriminating between AUD and HC is performed using SVM technique (Kaul and Raina, 2022; Roy and Chakraborty, 2023). The experiment is conducted for EC feature vectors generated at 64 frequency bands. Notably, each band is a bandwidth of (70–0.1) Hz/64 = 1.092 Hz. Figure 6 illustrates the method for evaluation of EC at all 64 frequency bands. This analysis serves as a supplementary investigation to gain additional insights on the PDCaM for classification of AUD vs. HC. Subsequently, the matrices for each frequency bands across all subjects were combined, resulting in a final matrix with dimensions equal to 4, 380 × 324 and 4, 827 × 324 feature vectors for AUD and HC, respectively.

SVM is chosen for its effectiveness in discerning different patterns or categories in data, as it excels in maximizing the margin between classes, thereby enhancing its ability to generalize well to unknown data. Hence, SVM is frequently used in several fields, such as text recognition (Kaur and Singh, 2023), natural language processing (Hussain et al., 2023), and medical signal or image classification (Asri et al., 2020; Verma and Sharan, 2023; Ali et al., 2022). Here, the SVM parameters are obtained via Bayesian optimization to ensure the best SVM is utilized in classification of the EC feature vector. This process aimed to identify the most suitable model parameters, including the kernel function, scale, and box constraints, for training classifiers for all 64 frequency bands.

Evaluation across the 64 frequency bands aimed to explore the distinctiveness of the AUD and HC data across the different frequency bands and determine the extent to which it contributes to classification accuracy. The performance of the SVM model was evaluated based on the obtained accuracy values, providing insights into the effectiveness of using the 64 frequency bands for AUD classification.

## 4 Result and discussion

The result of this study is presented as follows. Firstly, Section 4.1 visualizes the Partial-Directed Coherence matrices of one subject with AUD and one HC subject, across 4 different frequency bands. The initial exploration involves employing SVM in Section 4.2 to evaluate the discriminative capacity of the 19-channel PDCaM through classification based on 64 frequency bands. Then, the performance of the top-5 accuracy from the 64 bands is further evaluated, using confusion matrix and ROC plots. Lastly, the classification using brain rhythm is investigated and compared with a previous study.

### 4.1 Scatter plot of PDC adjacency matrix feature vectors

**Figure 7:**
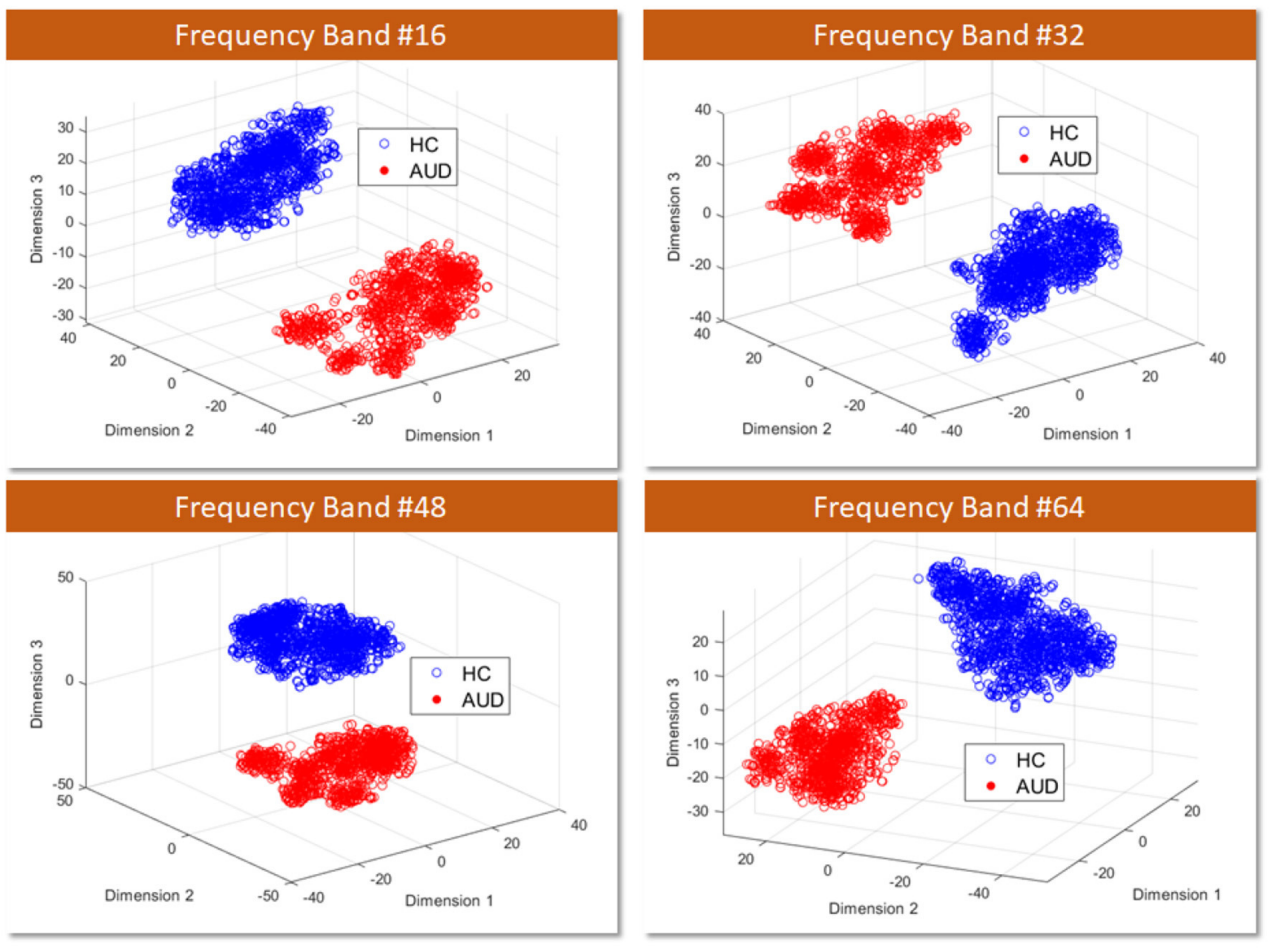
3D visualization of AUD and HC PDCaM feature vectors using t-SNE technique, selected for 4 frequency bands; 16, 32, 48, and 64.

Firstly, to assess the distinguishability of AUD and HC using PDCaM data, a 3D scatter plot is employed. Figure 7 displays a 3D scatter plot generated using t-distributed Stochastic Neighbor Embedding (t-SNE) to visualize the feature vectors distribution of AUD and HC subjects. The plots are shown at 4 different frequency bands out of the 64 bands, showing the first three main features extracted via the t-SNE technique.

T-SNE is a technique employed for dimensionality reduction, facilitating the visualization of high-dimensional data in a lower-dimensional space, commonly 2D or 3D. It proves advantageous in capturing intricate patterns within complex datasets, as it conserves the local structure and relationships among data points. From Figure 7, the scatter plot illustrates the first three main features extracted via the t-SNE technique. It is evident that the two classes of AUD and HC are distinguishable based on the cluster plot.

To further explore this analysis, a machine learning algorithm called SVM is employed to assess the accuracy of AUD detection for each frequency band. SVM provides valuable insights into the classification performance and helps understand how well the algorithm can distinguish between the two classes based on frequency-band PDCaM data.

### 4.2 Classification of AUD vs. HC PDC adjacency matrix using SVM

This section outline the evaluation of PDCaM in EEG recordings in the following manner: initially, the classification is conducted across all 64 frequency bands; subsequently, a thorough assessment is performed focusing on the top 5 frequency bands, and finally, an evaluation is carried out based on 5 distinct brain rhythms.

#### 4.2.1 Classification of AUD vs. HC using 64-band PDC adjacency matrix

Here, the PDCaM will be examined based on different frequency bands to explore its potential in the classification of AUD vs. HC across different frequency ranges. This analysis will provide valuable insights into the effectiveness of using frequency-specific PDCaM data for distinguishing between AUD and HC.

**Figure 8:**
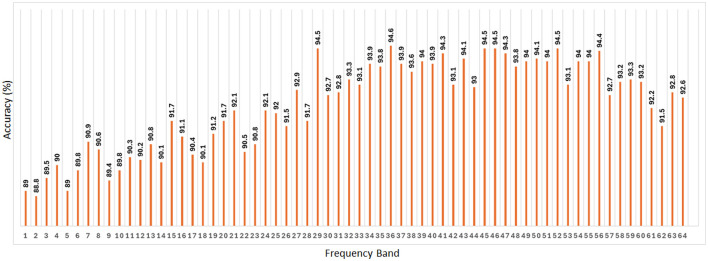
Classification accuracy of SVM across all 64 frequency bands taking PDC adjacency matrix as its input. The average value of the accuracy is 92.3%.

As presented in Figure 8, the accuracy of classification using SVM remains consistent across the 64 frequency bands, exhibiting an average accuracy of 92.3%. Additionally, Figure 8 shows the best accuracy of 94.6% using band 36 and the other 4 best accuracy of 94.5% are from band 29, 45, 46, and 52. These findings suggest that the SVM algorithm performs reliably in classifying AUD across various frequency bands, with the accuracy remaining stable.

#### 4.2.2 Evaluation of the top-5 frequency bands

**Figure 9:**
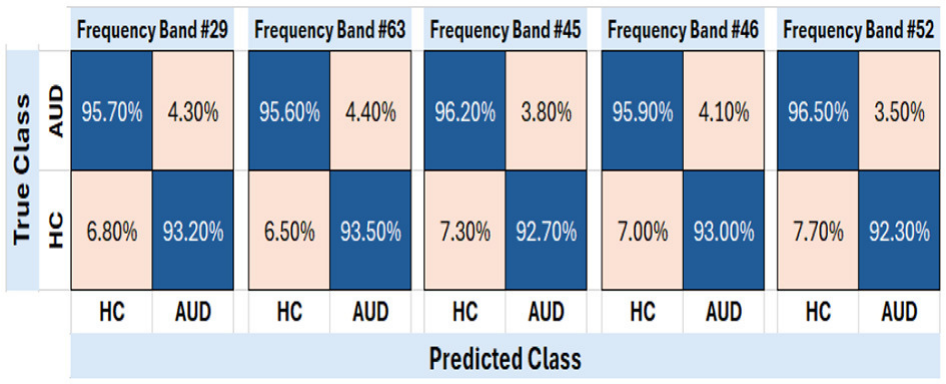
Confusion matrix of test PDCaM for the top-5 frequency bands; 29, 36, 45, 46, and 52. The average accuracy for these bands is 94.5%.

**Figure 10:**
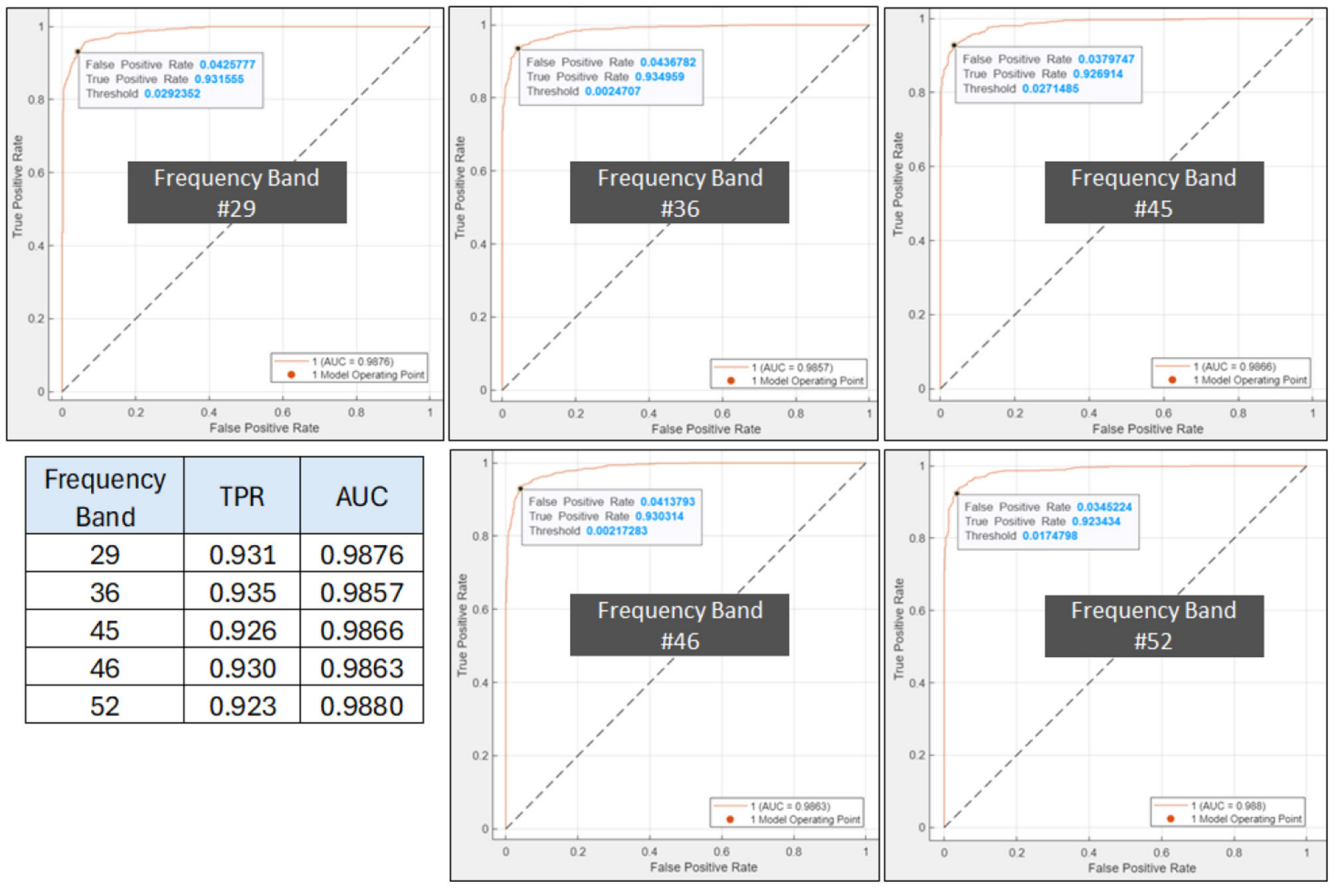
ROC plot for AUD indicating the SVM's optimal operating point, TPR and AUC top-5 frequency bands; 29, 36, 45, 46, and 52. The inset table lists the TPR and AUC values for the 5 bands.

For better insight on the performance of the top-5 frequency band as determined from the previous section, the PDCaM is further trained and tested on SVM to generate the confusion matrix and the ROC, as shown in Figures 9, 10. The hyperparameters of the SVM are obtained via Bayesian optimization. Notably, the SVM for the 5 bands utilized Gaussian as its kernel except for band 46 which is polynomial type.

The plot of the confusion matrix as depicted in Figure 9 provides a comprehensive assessment of the classification results by illustrating the number of correctly classified instances and the misclassifications for each class. The results indicate that the trained SVM model, utilizing PDCaM as its input, demonstrates a high level of accuracy in classifying AUD. The model achieves an impressive accuracy rate of 94.5%, highlighting its effectiveness in accurately distinguishing between individuals with AUD and HCs. Notably, the average AUC of all 5 frequency bands observed in Figure 10 is >0.98 for both classes indicating that the developed model exhibits strong performance in distinguishing between HC and AUD.

Moreover, the ROC plots in Figure 10 show the optimal operating points for the trained SVM, generated for AUD as the positive class. For AUD classification, the model's optimal operating point lies at true positive rate (TPR) values between 0.923 to 0.935. The obtained results demonstrate that the classifier has achieved an impressive TPR, accurately identifying a significant number of positive samples representing instances belonging to the AUD. The FPR is alternatively known as 1-specificity and signifies the proportion of negative samples that are incorrectly classified as positive. Additionally, as FPR values are significantly low, it indicates that the classifier performs exceptionally well by not misclassifying any negative samples as positive, thus demonstrating its outstanding performance. These findings suggest that the classifier effectively distinguishes between positive and negative samples and possesses a high likelihood of correctly identifying positive cases.

#### 4.2.3 Classification of PDC adjacency matrix across 5 brain rhythms

Lastly, the accuracy of AUD detection is further analyzed in terms of 5 different brain rhythms. Table 1 presents the classification accuracy achieved using PDCaM across various brain rhythms, alongside their corresponding frequency ranges and frequency bands. The table further compares these accuracy values with those obtained from a previous study that employed the same dataset (Khan et al., 2021c). However, the previous study employed a 3D CNN instead of the SVM that is used in this study.

**Table 1:** Comparison of accuracy across brain rhythms: current study vs. previous study (Khan et al., 2021c).

			**Accuracy (%)** ± **standard deviation**	
**Brain rhythm**	**Frequency range (Hz)**	**No. of frequency band**	**Current study**	**Previous study**
Delta	0–4	1–6	91.00 ± 0.57	77.05 ± 6.97
Theta	4–8	7–12	92.43 ± 0.59	78.83 ± 7.94
Alpha	8–12	13–19	93.10 ± 0.59	81.27 ± 6.66
Beta	12–30	20–47	95.93 ± 0.47	86.16 ± 5.26
Gamma	30–40	48–64	96.27 ± 0.45	83.44 ± 5.51

The results in Table 1 demonstrate that all brain rhythms of the current study exhibit high accuracy in classifying AUD. The gamma band provides the highest accuracy, followed by alpha and theta. However, the lower frequency bands, delta and theta, exhibit lower accuracy. This observation could be attributed to various factors, such as the complexity of AUD-related brain activities in those frequency ranges. Further investigations are required to gain deeper insights into the reasons behind this phenomenon.

On the other hand, the accuracy of brain rhythms in the previous study is lower than the accuracy achieved in the current study. Although the highest brain rhythm, beta, had the highest accuracy in the previous study, its performance is surpassed by all brain rhythms in the current study. The comparison enables observation of performance differences between the machine learning and deep learning architectures in classifying AUD based on PDCaM for different brain rhythms. These findings highlight the effectiveness and potential advantages of the developed algorithm for AUD detection using PDCaM, particularly in frequency-based classification with SVM.

### 4.3 Classification of reduced PDC adjacency matrix across 5 brain rhythms

**Figure 11:**
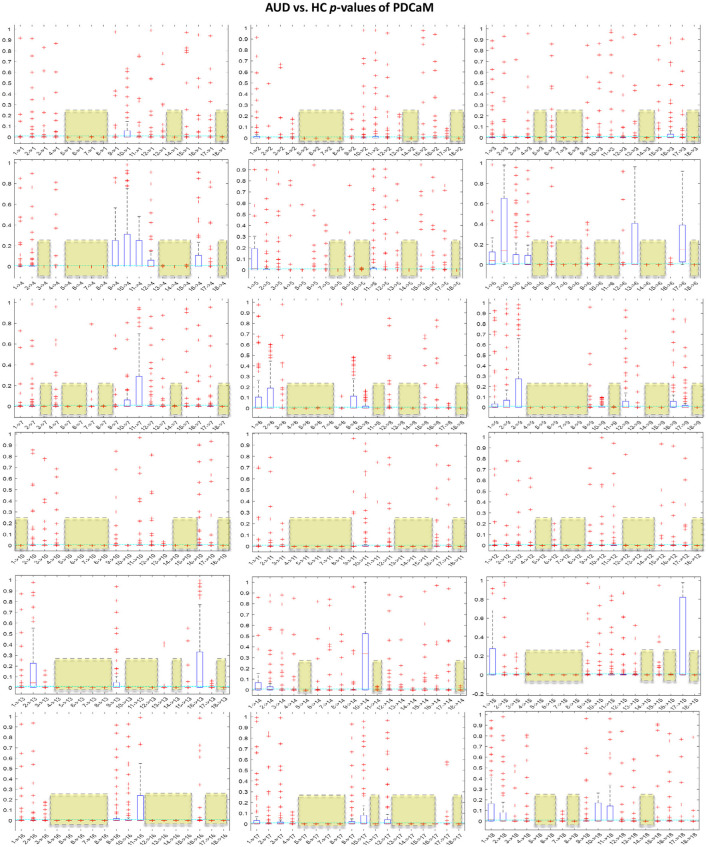
Boxplot of *p*-values evaluated for all 18 × 18 connectivities of the PDCaM. The yellow boxes highlight the connectivies with *p*-value < 0.01, i.e., 99.9% significance in discriminating between AUD vs. HC. The number of connectivities with *p*-value < 0.01 (yellow boxes) is 147. The electrodes are labeled numerically from 1 to 18, corresponding to the following electrode positions: FP1, FP2, F3, F4, C3, C4, P3, P4, O1, O2, F7, F8, T3, T4, T5, T6, FZ, and PZ, respectively.

It is noteworthy to emphasize that employing a greater number of feature vectors, such as in the context of classification with the five brain rhythms, results in improved accuracy. However, this comes at the cost of a significant increase in the length of the feature vector. Specifically, for the beta and gamma bands, the feature vector length expands to 5,509. Training the SVM on such a large feature vector will require large memory and computational power. In an effort to reduce the feature vector of PDCaM, a statistically significant test using the ANOVA technique is employed. The plot of AUD vs. HC *p*-values for all 18 × 18 connectivites are given in Figure 11.

**Figure 12:**
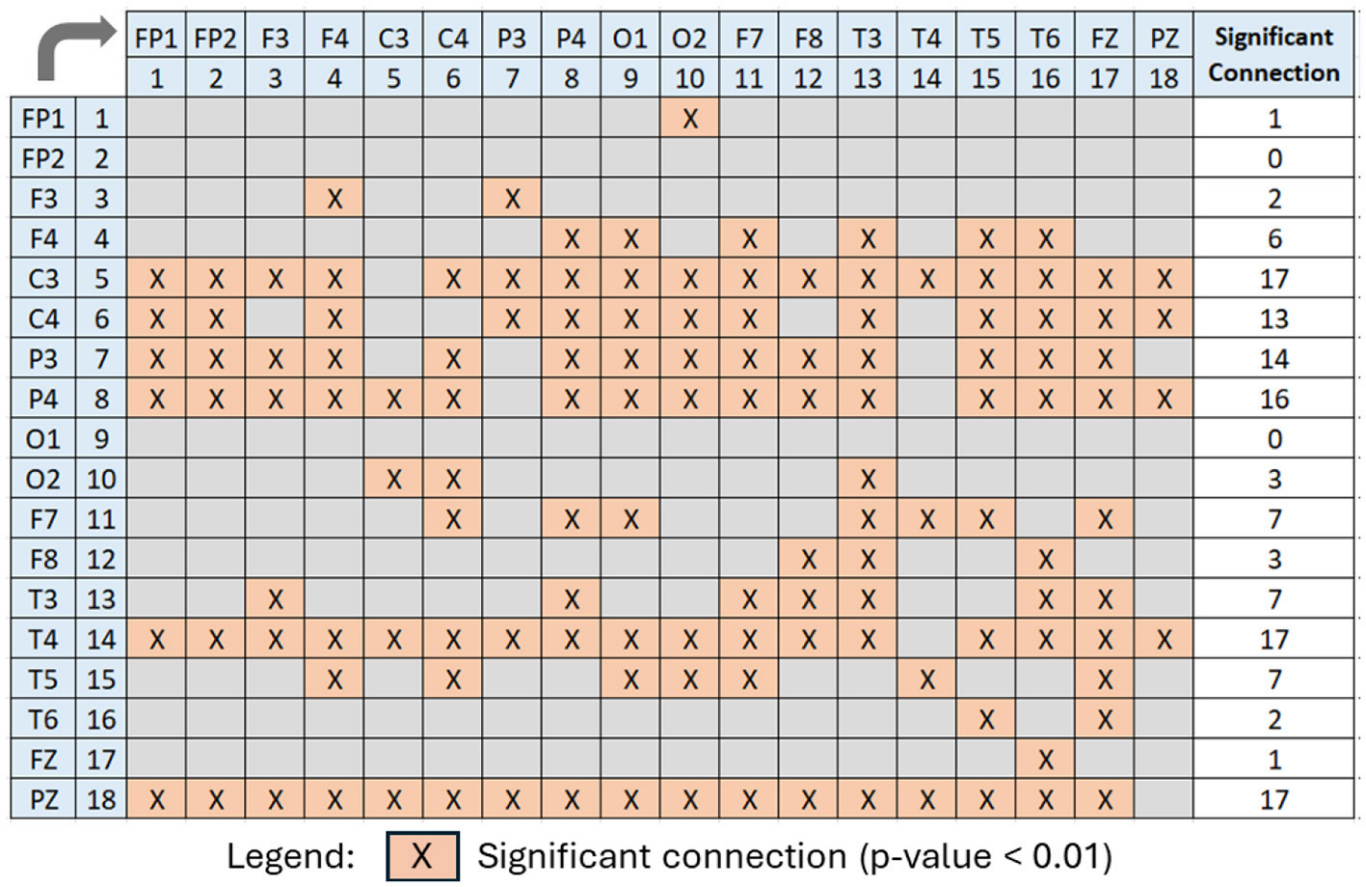
Significant PDCaM connections (*p*-value < 0.01) among all 18 electrodes, extracted from Figure 11, for further analysis of affected brain functions in AUD subjects. The arrow denotes the direction of connectivity.

For further analysis of Figures 11, 12 has been generated to identify significant connections and their relationship to brain functions. Consistent with a prior research (Khan et al., 2021c), four electrodes–C3, C4, P3, and P4–exhibit significant connectivity with at least 13 other brain nodes. This finding aligns with the known impact of alcohol on the brain's communication pathways, which disrupt the brain's functionality. The electrodes C3 and C4, positioned over the left and right central areas of the brain, respectively, capture activity from the primary motor cortex and the somatosensory cortex. These regions play a critical role in motor planning, execution, and sensory processing. Similarly, electrodes P3 and P4 are associated with cognitive processing, integral to the processing of vestibular and proprioceptive information. The significant connectivity of these electrodes underscores the widespread functional disruptions caused by alcohol, particularly in regions essential for motor and cognitive integration.

**Figure 13:**
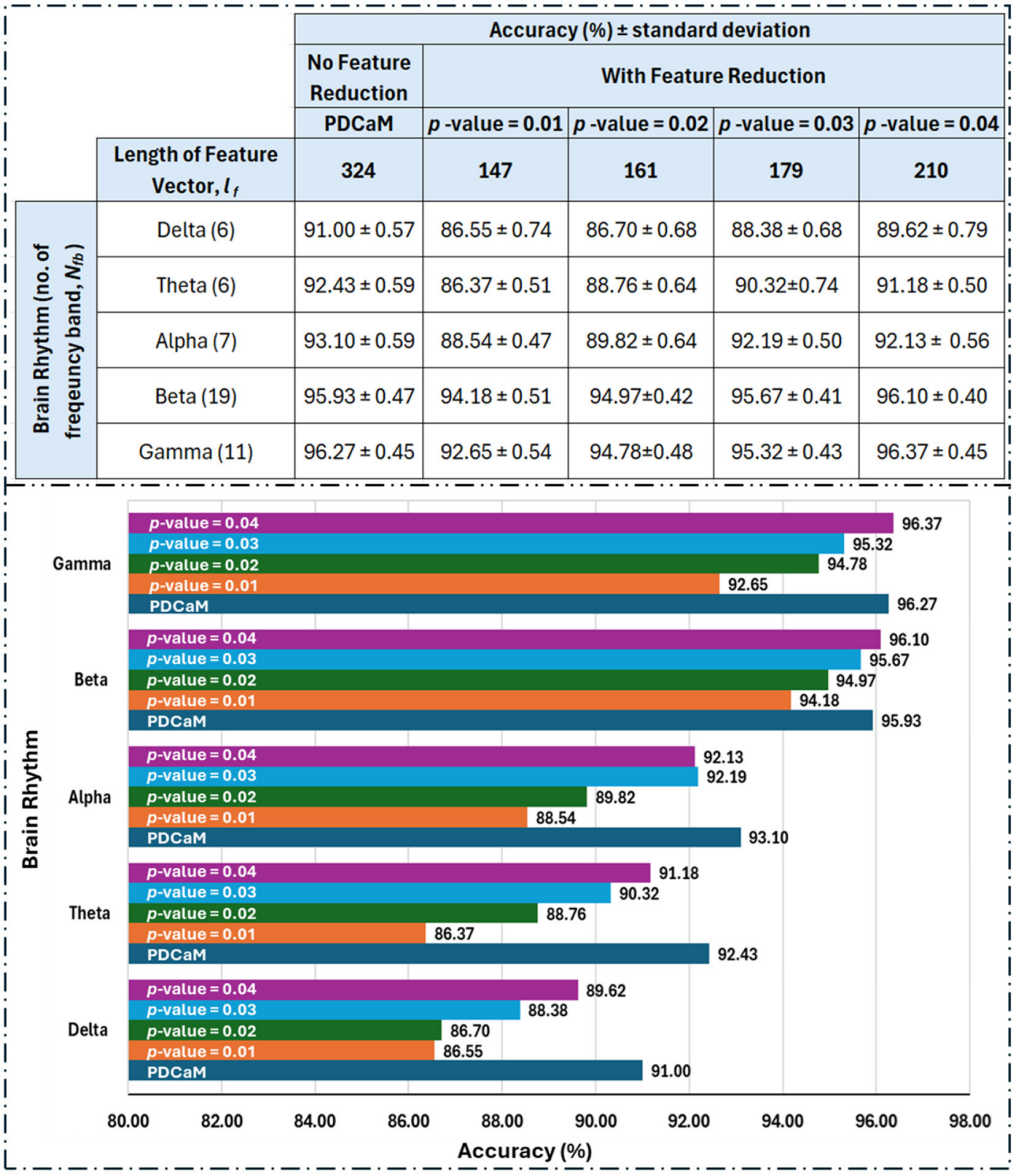
Tabulated classification accuracy evaluated for reduced feature vectors with *p*-value between 0.01 to 0.04 **(top)** and its corresponding barplot **(bottom)**. The length of feature vector for the original PDCaM is 324 and this value is reduced to between 147 to 210 as the *p*-value is increased from 0.01 to 0.04. The number in bracket in the 1st column of the table **(left)** indicate the number of frequency bands as per listed in Table 1. The length of the feature vector for each cases is *N*_*fb*_×*l*_*f*_.

We further evaluate the classification accuracy as we reduced the feature according to the *p*-value and the results are tabulated and plot in Figure 13. Based on the table shown in Figure 13, clearly utilization of full 324 connectivities has resulted in the best accuracy across all 5 brain rhythms but not in the case of beta and gamma. Notably, at a *p*-value of 0.04, which corresponds to a 96% confidence level, the accuracies for beta and gamma exceed those achieved with the full-length feature set. This trend is anticipated since the best accuracies with the full feature set are 95.93% and 96.27% for beta and gamma, respectively. If the investigation into feature reduction continues for *p*-values greater than 0.04, we may expect better accuracies than those of the full feature set for delta, theta, and alpha to occur at *p*-values of 0.09, 0.08, and 0.07, respectively. However, selecting higher *p*-values is not desirable as it increases the feature vector length and may degrade the performance of the classifier.

Based on the feature reduction experiment, gamma demonstrated a significant advantage by achieving the highest accuracy of 96.37% while using only 64.8% (210 features) of the full 324-length feature set. This accuracy is slightly higher compared to its performance with the full feature length.

## 5 Conclusions

In conclusion, this research paper presents a new approach for AUD detection by utilizing PDCaM of resting-state EEG signals and SVM classifier. The investigation is rooted in the understanding that abnormal brain patterns are linked to neurological disorders, making PDCaM a promising means to represent brain networks in various subjects. The derived EC is based on the PDC from EEG recordings of 19-channel EEG recording, producing 18 × 18 PDCaM, since Cz is used as the reference node. Then, the proposed EEG classification algorithm incorporates PDCaM and SVM to accurately classify AUD subjects from HCs. By analyzing the features of EEG signals from the AUD dataset, the algorithm's development for AUD detection is systematically achieved. Notably, the PDCaM is transformed into a row vector of length 324 features to facilitate the training and testing of the SVM model.

The AUD classification using PDCaM and SVM is tested across 64 frequency bands achieving an impressive accuracy of 94.6% and a high AUC of >0.98. This shows that the proposed PDCaM+SVM classification model has a high TPR while maintaining a low FPR. In other words, the model is effective in correctly identifying positive cases while minimizing the misclassification of negative cases.

Furthermore, a brain rhythm-based AUD detection is conducted across 5 different bands using SVM, revealing a high accuracy between 91% to 96.27%. Notably, the gamma bands exhibited the highest accuracy among the analyzed brain rhythms.

The proposed PDCaM-based approach for detecting AUD using resting-state EEG signals and SVM classifiers demonstrates significant potential for clinical application. Achieving high accuracy (94.6%) and AUC (>0.98), the model shows promise as a reliable, non-invasive diagnostic tool for distinguishing AUD subjects from healthy controls. Its ability to analyze specific brain rhythms, particularly the gamma band, highlights its potential for both early detection and monitoring of AUD-related neurophysiological abnormalities.

For real-world implementation, several challenges must be addressed. Scaling the methodology to larger and more diverse populations is crucial to validate its robustness and generalizability. Additionally, computational demands arising from high-dimensional PDCaM feature vectors require optimization through efficient algorithms and feature selection to enable deployment on portable or edge devices. Automating the pipeline from EEG preprocessing to classification is another critical step to facilitate its integration into clinical workflows.

Broader implications of this study include its applicability to monitoring treatment efficacy and its potential extension to other neurological or psychiatric disorders with similar EEG abnormalities. By addressing scalability, computational efficiency, and automation, the findings of this research pave the way for impactful applications in diagnosing and managing AUD. This could ultimately improve patient outcomes through targeted interventions and personalized care strategies.

## Data Availability

The data analyzed in this study is subject to the following licenses/restrictions: the data used in this study has been acquired from CISIR's data repository. It will be made available for use upon reasonable request from the corresponding author after signing a formal data sharing and usage agreement. Requests to access these datasets should be directed to Norashikin Yahya, norashikin_yahya@utp.edu.my.
